# Chromatin Dynamics in Intestinal Epithelial Homeostasis: A Paradigm of Cell Fate Determination *versus* Cell Plasticity

**DOI:** 10.1007/s12015-020-10055-0

**Published:** 2020-10-13

**Authors:** Jérémie Rispal, Fabrice Escaffit, Didier Trouche

**Affiliations:** LBCMCP, Centre of Integrative Biology (CBI), Université de Toulouse, CNRS, UPS, Toulouse, 31062 France

**Keywords:** Intestinal epithelium, Homeostasis, Stem cell proliferation, Differentiation, Epigenetics, Chromatin remodeler, Histone post-translational modification, Lineage commitment, Response to environment

## Abstract

The rapid renewal of intestinal epithelium is mediated by a pool of stem cells, located at the bottom of crypts, giving rise to highly proliferative progenitor cells, which in turn differentiate during their migration along the villus. The equilibrium between renewal and differentiation is critical for establishment and maintenance of tissue homeostasis, and is regulated by signaling pathways (Wnt, Notch, Bmp…) and specific transcription factors (TCF4, CDX2…). Such regulation controls intestinal cell identities by modulating the cellular transcriptome. Recently, chromatin modification and dynamics have been identified as major actors linking signaling pathways and transcriptional regulation in the control of intestinal homeostasis. In this review, we synthesize the many facets of chromatin dynamics involved in controlling intestinal cell fate, such as stemness maintenance, progenitor identity, lineage choice and commitment, and terminal differentiation. In addition, we present recent data underlying the fundamental role of chromatin dynamics in intestinal cell plasticity. Indeed, this plasticity, which includes dedifferentiation processes or the response to environmental cues (like microbiota’s presence or food ingestion), is central for the organ’s physiology. Finally, we discuss the role of chromatin dynamics in the appearance and treatment of diseases caused by deficiencies in the aforementioned mechanisms, such as gastrointestinal cancer, inflammatory bowel disease or irritable bowel syndrome.

**Figure:**
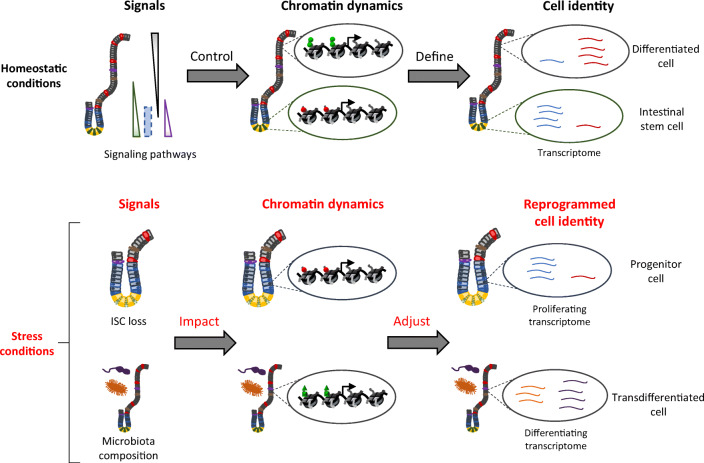
Graphical abstract

Graphical abstract

## Introduction

The intestinal epithelium is entirely renewed every 3 to 5 days. This renewal is mediated by Intestinal Stem Cells (ISCs), which reside at the bottom of proliferative compartments, called “crypts” [1, 2]. The ISCs divide to self-renew or to give rise to progenitor cells, which in turn undergo several cycles of division before committing to secretory or absorptive lineages [3]. Then, cells migrate along the villi, and acquire terminal differentiation features. When cells reach the top of the villi, they detach and die by anoikis, a detachment-induced apoptotic process. All these phenotypic shifts are associated with major changes in gene expression [4]. Indeed, about 4000 to 6000 genes are differentially expressed in crypts compared to villus cells [5, 6].

The fine regulation of this renewal allows the intestinal epithelium to reach a crucial equilibrium, called “homeostasis”, which is required for essential functions of the tissue, such as being a barrier, or an immune or metabolic regulator. This homeostasis, which has been extensively studied during the last decades, is controlled by signaling pathways [7], such as Wnt which is essential for the proliferation and stemness maintenance of crypt cells. Decrease in Wnt activity is required for differentiation processes occurring along the villus [8–10]. This process also involves specific transcription factors (TFs) that directly regulate gene expression. For example, the homeobox transcription factor CDX2 is the master regulator of intestinal cell identity [11, 12]. Indeed, CDX2 inhibits proliferation of intestinal cells, as shown in different species [13, 14], and controls the transcriptional program associated with intestinal differentiation [15]. The role transcription factors play depend on cell type and are dictated through their dynamic interaction with gene regulatory regions. For example, CDX2 interacts with 10,733 new sites during differentiation of ISC, whereas only 1372 of interaction sites are shared between ISC and villus cells *in vivo* [6]. Although regulatory mechanisms influencing TF dynamics are poorly understood, they are not due to differential expression of the TF encoding genes, which is actually similar between cell types [15–17]. Shivdasani’s and Beaulieu’s laboratories have hypothesized that chromatin dynamics could govern TF recruitment and thereby, be a major player in the regulation of epithelial intestinal homeostasis [16, 18]. Moreover, chromatin dynamics, itself modified by TF recruitment, could play a role upstream or downstream of signaling pathways involved in homeostasis.

Chromatin is a highly dynamic structure involving the association between DNA and proteins (histones and non-histones). Wrapping 146 bp of DNA around a histone octamer (2 H2A/H2B dimers and 1 H3/H4 tetramer) gives rise to a nucleosome, the basic unit of chromatin [19]. Chromatin is essential to regulate processes occurring at the DNA level, such as transcription [20]. A wide range of modifications participate in chromatin dynamics and in regulating these processes: post-translational histone modifications, histone variant incorporation, ATP-dependent remodeling, DNA methylation, non-coding RNA expression, etc. Most of these modifications are reversible. They can be deposited on and removed from chromatin by specific enzymes (writers *versus* erasers), allowing dynamic regulation of these marks. Some modifications can directly affect chromatin structure, others function as signaling events. Their presence or absence is recognized by proteins (readers) containing specific domains, such as bromodomains, PHD domains, chromodomains, etc. …, which mediate regulation of DNA-linked processes, like transcription.

The role of chromatin modification in cell fate has been extensively studied in embryonic stem cell maintenance and differentiation (reviewed in [21–23]). Recent advances indicate their importance in the homeostasis of adult tissues, like intestinal epithelium. In this review, we focus on the role of chromatin dynamics in the control of epithelial intestinal homeostasis.

## Role of Chromatin Modification in Intestinal Cell Fate Regulation

**Fig. 1:**
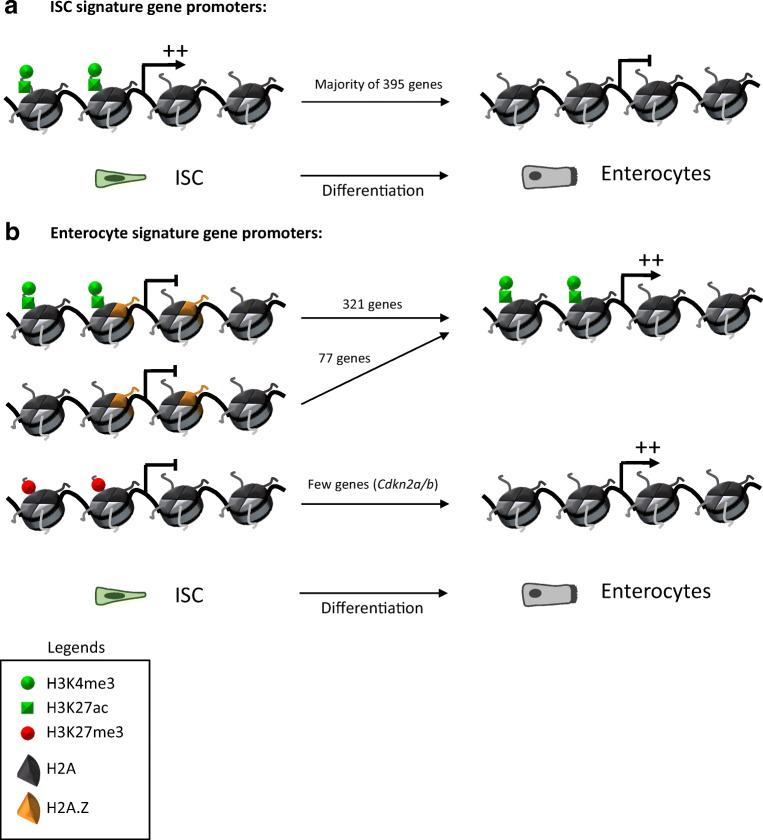
Dynamics of chromatin modification during ISC differentiation. **A** At the promoter of ISC signature genes, main changes in chromatin modification during enterocytes differentiation are loss of H3K4me3 and H3K27ac, correlated with loss of gene expression. **B** At the promoters of approximately 400 enterocyte signature genes, H2A.Z is lost during differentiation, correlating with a gain in gene expression. Noted that H3K4me3 and H3K27ac are either already present, or added to these promoters. Moreover, there is also a loss of H3K27me3 at the promoters of a few enterocyte signature genes, such as *Cdkn2a/b*

The dynamics of several chromatin modifications during specification and differentiation of ISCs, in association with transcriptional changes has recently been investigated [5]. The authors reported a decrease in both H3K4me3 and H3K27ac at promoters of most ISC signature genes (i.e. genes predominantly expressed in ISCs compared to enterocytes) during ISC to enterocyte differentiation (Fig. 1A). The loss of these two marks, known to correlate with active transcription [24, 25] and called “active marks” thereafter, is consistent with decreased expression of these genes during differentiation.

Kazakevych et al. also identified an important subset of 2142 genes whose expression increases during enterocyte differentiation [5]. Induction of the active marks H3K4me3 and H3K27ac during differentiation is observed at 71% of the promoters of these enterocyte signature genes. The repressive mark H3K27me3 decreases at the promoter of several genes, such as the cluster of cell cycle inhibitors *Cdkn2a/b* (INK4 locus). Another prominent change is the decreased presence of histone variant H2A.Z at the promoters of 421 of these genes (Fig. 1B). All these modifications, as well as their writers, erasers and readers, can thus participate in the control of epithelial intestinal cell identities.

### Histone Variant H2A.Z

H2A.Z, a histone variant of the canonical H2A [26], is mostly enriched at transcriptional regulatory regions, i.e., TSS and enhancers [27, 28]. In mammals, it is mostly incorporated by two ATP-dependent remodeling complexes relying on p400 or SRCAP enzymatic activity [29, 30]. H2A.Z is synthetized as three isoforms (H2A.Z.1, H2A.Z.2 and H2A.Z.2.2) encoded by 2 different genes *H2AFZ* and *H2AFV* [31]. This histone variant plays a major role in transcriptional regulation, having either a positive or negative role, depending on gene or cell type [28, 29, 32]. However, this differential effect seems to be dependent on its post-translational modification, since its acetylation correlates with positive effects on gene expression [33, 34], and its ubiquitination is linked to negative transcriptional effects [35].

H2A.Z plays an essential role in the maintenance of intestinal epithelium integrity and its constant renewal. Rispal et al. demonstrated that H2A.Z.1 (the most studied isoform) is important for the proliferation of intestinal cells [36]. Zhao and collaborators showed that deletion of both H2A.Z isoforms (H2A.Z.1 and H2A.Z.2) leads to severe morphological changes of the intestinal epithelium, resulting in important weight loss in mice. Moreover, the authors showed that deletion of Znhit1, a subunit of SRCAP complex, leads to the same phenotype, suggesting that H2A.Z mediates the effect of SRCAP complex in this context [37]. The role in this process of p400, the other H2A.Z-incorporating enzyme, was not considered in this study and thus remains to be investigated.

**Fig. 2:**
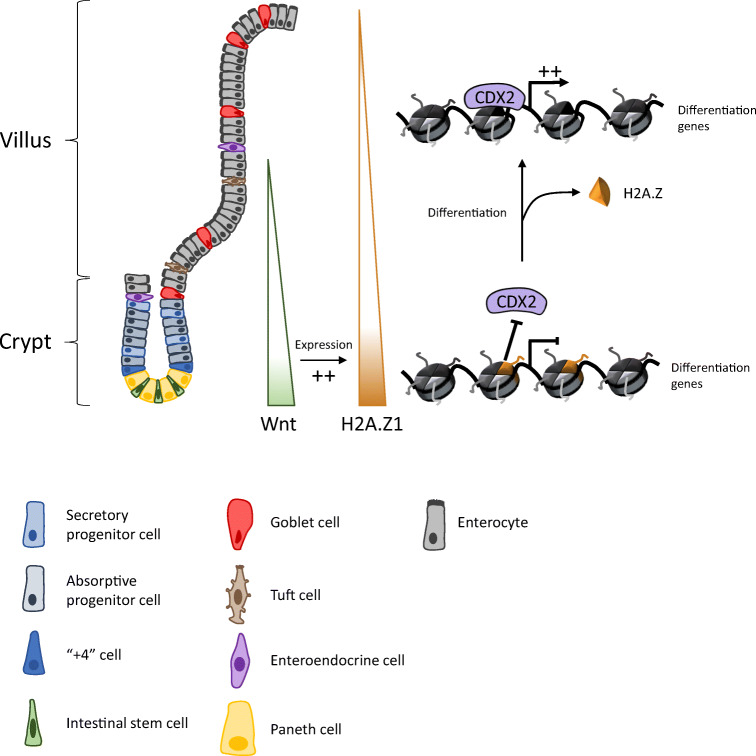
Model for H2A.Z1’s role in the maintaining proliferative abilities and precluding differentiation. In the crypt, high Wnt activity favors the expression of the H2A.Z1 encoding gene (*H2afz*). Thus, H2A.Z1 participates in cell proliferation, and is also incorporated at the promoters of differentiation genes, where it inhibits the binding of the transcription factor CDX2 and thereafter gene expression. During cell migration along the villus, reduction in Wnt activity leads to a decrease in H2A.Z1 expression. This variant is then removed from the chromatin, allowing CDX2 binding and the expression of CDX2-target differentiation genes

H2A.Z is also important for the control of intestinal differentiation [36]. Rispal et al. demonstrated the inhibitory role of H2A.Z.1 on the expression of differentiation genes specific for enterocytes (*SI, LPH*) and for goblet cells (*MUC2, MUC4*) *in cellulo* and *in vivo*. H2A.Z.1 inhibits binding of the transcription factor CDX2 at the promoter of its target genes (Fig. 2). Moreover, the authors showed that the Wnt pathway positively regulates expression of the H2A.Z.1-encoding gene. The authors proposed that the high amount of H2A.Z.1 in the crypt, probably due to high Wnt activity in the stem cell niche, allows cell proliferation and inhibits differentiation. During terminal differentiation, decrease of Wnt activity leads to down-regulation of H2A.Z expression, thereby allowing expression of differentiation genes thanks to increased CDX2 binding [36].

Note that this study focused on the H2A.Z.1 isoform. Given the possibility of specific (or even opposite) effects of each H2A.Z isoform on transcription [32, 38–40], it would be valuable to investigate the role of the H2A.Z.2 and H2A.Z.2.2 isoforms in the ISC niche.

### Histone Acetylation Dynamics

Histone acetylation consists of adding an acetyl group to lysine residues on histones. By neutralizing the histone’s positive charge, acetylation decreases the interaction between DNA and histones [41], leading to a local opening of the chromatin. Due to this structural effect, histone acetylation mostly translates into transcriptional activation, by allowing binding of transcription machineries to DNA [42]. In addition, histone acetylation is also recognized by specific domains, such as bromodomains, and proteins containing such domains that function as “readers” of acetylated proteins. The presence of this modification is dynamically regulated by HATs (Histone Acetyl Transferases), which function as writers, and HDACs (Histone DeACetylases), the erasers. The family of HDAC enzymes is composed of 18 members, belonging to 4 distinct classes, depending on their function and sequence homology. Class I, II and IV are considered “classical” HDACs and have a zinc-dependent active site, whereas Class III (sirtuins) enzymes are NAD^+^-dependent proteins [43]. HATs are divided into 5 families, based on their structure, including GNATs (GCN5, PCAF…), MYST (Tip60…), p300/CBP, SRCs (nuclear receptor coactivators) and TAF1/CLOCK families [44]. HDACs and HATs have been mostly described as transcriptional repressors and activators, respectively, consistent with a positive role for histone acetylation in transcription [44, 43].

Many studies have addressed the role of histone acetylation in intestinal homeostasis by investigating the involvement of HDACs and HATs. Tou and colleagues showed that HDAC1 and HDAC2 are the most highly expressed HDACs in adult intestinal epithelium [45]. Moreover, these enzymes are more abundant in crypts than in villi of mice [46], suggesting their essential role in stemness and tissue renewal. Furthermore, KO (Knock-out) of both genes encoding for HDAC1 and HDAC2, leads to loss of stemness, characterized by decreased expression *in vivo* of ISC markers (*Lgr5* and *Olfm4*) and loss of ability to form 3D intestinal organoids [46]. This double KO also leads to the decrease of intestinal cell proliferation [46], and this phenotype is also observed in other studies using HDAC inhibitors SAHA [47] or β-hydroxybutyrate (βHB, [48]). Altogether, these results confirm the essential role of HDACs in maintaining the crypt compartment. Although the mechanisms are not fully understood, increased expression of the cell cycle inhibitor p21 upon HDAC inhibition suggests that the p21-encoding gene is probably a major target of these HDACs to prevent premature differentiation and cell cycle arrest-dependent gene expression [47, 48].

In accordance with this hypothesis, pharmacological HDAC inhibition leads to increased expression of differentiation marker genes *in cellulo* and *in vivo*. SAHA treatment upregulates enterocyte-specific genes, such as *Sucrase-Isomaltase*, in Caco-2/15 cells (an *in vitro* model of enterocyte differentiation) and in mice [47]. This result is consistent with the increase of H3K27ac on the promoters of enterocyte signature genes during enterocyte differentiation observed in Kazakevych et al. [5] (see Fig. 1B), although the authors did not investigate whether H3K27ac was increased upon SAHA treatment. HDAC inhibition with βHB treatment leads to the increase of markers of several differentiation lineages: enterocytes, goblet cells and Paneth cells [48]. The HDAC responsible for this effect remains unidentified. Of note, Zimberlin and colleagues did not observe any increase in differentiation marker expression in *Hdac1* and *Hdac2* KO animals [46], indicating that these two highly expressed HDACs are probably not involved. It will thus be of interest to investigate the function of other members of the HDAC family.

**Fig. 3:**
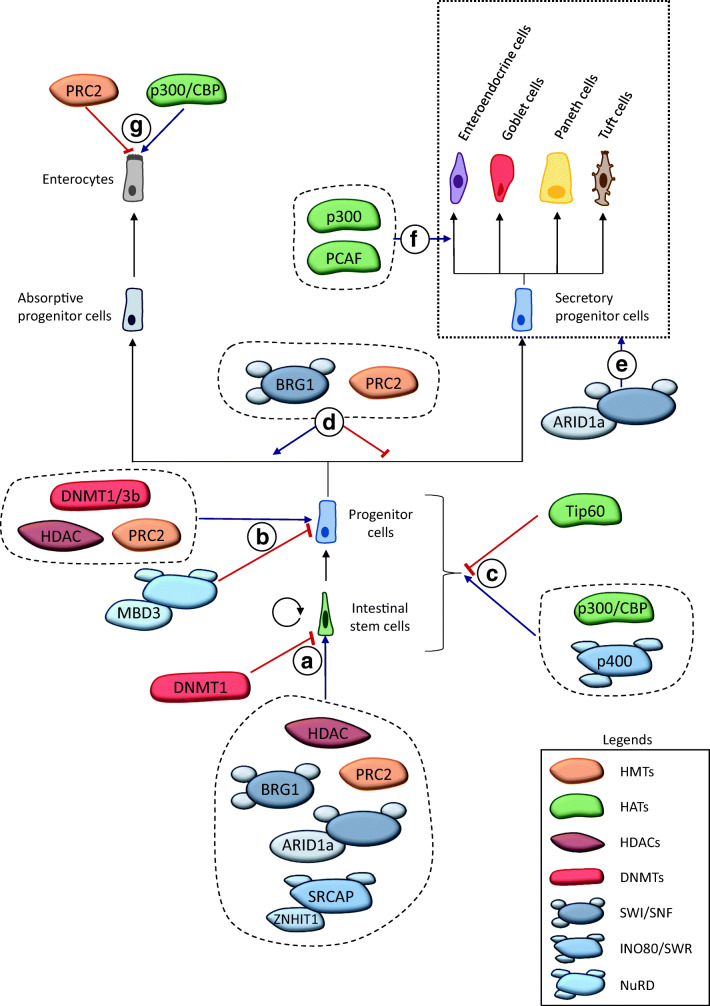
Role of chromatin modifiers in intestinal epithelial homeostasis. **A** **In stemness**. Many chromatin modifiers are essential for ISC maintenance. Deletion of PRC2, HDAC1/2 or ZNHIT1-containing SRCAP complex leads to loss of ISC and greatly impairs formation of organoids in 3D culture conditions. SWI/SNF complexes are also involved in this process, since ARID1a-containing complex promotes *Sox9* expression and since BRG1-containing complex directly favors *Lgr5*, *Olfm4* and *Ascl2* expression. DNMT1 is involved in restricting stemness by inhibiting expression of stem cell markers like *Olfm4, Sox9* and *Msi1*. **B** **Progenitor identity**. PRC2 and HDAC are essential for maintaining undifferentiated features of progenitor cells. Indeed, both modifiers favor cell proliferation through *Cdkn2a (Ink4)* inhibition and *p21* expression, and inhibit expression of differentiation genes. DNMT1 and DNMT3b are also involved in maintaining progenitor proliferation. In contrast, MBD3-containing NuRD complex is involved in restricting proliferative cells in the crypt compartment. **C** **Wnt pathway regulation**. Tip60, by suppressing β-CATENIN acetylation, inhibits proliferation of intestinal cells, whereas the p400 complex has the opposite effect, by promoting expression of Wnt target genes. CBP and p300 HATs promote expression of Wnt target genes by recruiting β-CATENIN/TCF4 transcription factors. **D** **Lineage choice**. PRC2 and SWI/SNF complex containing BRG1 help determine specific lineage during progenitor cell maturation. Indeed, both complexes inhibit commitment to the secretory lineage by repressing *Atoh1*/*Math1* expression, which promotes the absorptive fate. **E** **Secretory differentiation**. SWI/SNF complex containing ARID1a is also involved in secretory lineage differentiation. Indeed, although the temporal and spatial requirements of this complex for this lineage differentiation are unknown, all secretory cells are lost in the KO mice. **F** **Enteroendocrine differentiation**. The two HATs, p300 and PCAF, are involved in differentiation of enteroendocrine cells, by promoting expression of NEUROD1 target genes, especially *Secretin* gene. **G** **Enterocyte differentiation**. p300/CBP seems to be involved in enterocyte differentiation. Indeed, recruitment of these 2 HATs is correlated with an increase in expression of the CRBPII coding gene, which is involved in Vitamin A transport and metabolism in enterocytes. In contrast, PRC2 complex is involved in repressing enterocyte differentiation by inhibiting expression of genes like *SI, LPH* or *ALPI*

Altogether, these results show the essential role of HDAC in intestinal epithelium, especially for the maintenance of cells in an undifferentiated state (Fig. 3A and B).

The analysis of acetylation writers, HATs, showed that these enzymes are important for intestinal homeostasis by functioning as transcriptional coactivators for specific transcription factors. The two HATs, p300 and CBP, are highly expressed all along the adult intestinal epithelium, suggesting their involvement in establishing or maintaining the identity of many cell types [49] (Fig. 3C, F and G). Another study showed that, in rat models, p300 and CBP are more expressed just after birth [50]. This increase allows recruitment of these enzymes to promoters of the CRBPII (also known as RBP2, Retinol Binding Protein 2) encoding gene, favors local histone acetylation, and increases the binding of RNA Pol II. This result suggests the possible role of HAT dynamics in the increased expression of CRBPII after birth, essential for the transport and the metabolism of Vitamin A [50].

HATs are also important for the activity of the transcription factor NeuroD. NeuroD is crucial for enteroendocrine cell differentiation, especially by promoting the expression of Secretin [51, 52]. NeuroD is involved in targeting HATs p300 [53] and PCAF [54] to the promoter of Secretin gene *in cellulo*. PCAF recruitment leads to H3K9 acetylation, RNA Pol II binding and Secretin expression [54]. These results suggest how the two HATs, by acting as NeuroD coactivators, setup enteroendocrine lineage differentiation.

Another study also revealed the role p300 and CBP play in modulating two TCF4/β-Catenin targets (*Survivin* and *c-Myc*) *in cellulo* [55], *via* histone acetylation at these promoters but also by promoting acetylation of β-Catenin itself, which causes its stabilization and increases its transactivation abilities [56, 57]. In this context, interaction between p300/CBP and TCF4/β-Catenin causes not only the transcription factor recruitment of HATs, but also positive regulation of the transcription factor. During differentiation, KLF4 could regulate this process by inhibiting the interaction between HATs and TCF4/β-Catenin [58]. These results suggest that some HATs are critical downstream effectors of the Wnt pathway. However, this is balanced by the action of other HATs, since Tip60, a HAT from the MYST family, has a negative effect on the Wnt pathway by inhibiting β-Catenin acetylation, and thus on cell proliferation *in vivo* [59] (Fig. 3C). These results suggest a complex network of regulation, by different HATs and their dynamics, which affects Wnt signaling and its decisive physiologic roles.

Finally, another study suggested a role for p300 in mediating activity of two transcription factors in the intestine, MYB and CREB [49]. The authors have used the mice model *Plt6*, in which the p300 sequence is mutated (Y630N) impairing the interaction between p300 and MYB [60]. This mutation leads to reduced proliferative cells (PCNA^+^) number and of *Lgr5* expression [49], a phenotype very similar to MYB hypomorphic mutation [61, 62]. These phenotypic similarities suggest a role of p300/MYB in maintaining proliferation and stemness, in which p300 acts as a MYB coactivator. The result in p300 *Plt6* mice also shows that CBP is unable to compensate for the p300 functional deficiency. The intestinal specific KO of the CREB encoding gene leads to decreased proliferative cell number, loss of stemness and increased enteroendocrine and Goblet cell numbers [49]. However, the direct link between these phenotypes remains to be demonstrated.

Altogether, these results demonstrate how HATs, by acting as transcriptional coactivators (Fig. 3C, F and G), control intestinal epithelial identity. However, most of published studies are correlative and obtained in *in cellulo* models. *In vivo* analyses of HAT functions by deleting HAT-encoding genes in intestinal epithelium, are necessary to confirm and clarify their roles in homeostasis.

### Polycomb Repressive Complex 2 and H3K27me3 Dynamics

One of the most studied chromatin marks involved in cell fate control is H3K27me3, and Polycomb Repressive Complex 2 (PRC2) functions as its specific writer. H3K27me3 is associated with transcriptional repression, by playing a scaffold role for repressor complexes (such as the PRC1 polycomb complex), or by inhibiting recruitment of activator complexes or deposition of the active mark H3K27ac [63].

Beaulieu’s lab, using various cellular models [64], was the first to study the role of PRC2/H3K27me3 in intestinal homeostasis. In Human Intestinal Epithelial Crypt-like (HIEC) cells, human progenitor cell model, depletion of SUZ12 (a subunit of the PRC2 complex) leads to a proliferation decrease. In two enterocyte differentiation cell models (Caco-2/15 and HIEC^HNF1α/CDX2^), PRC2 is involved in repressing, in a CDX2/HNF1α-dependent manner, differentiation marker genes expression (*SI, LPH, ALPI*). The authors hypothesize that PRC2 could be essential for preserving progenitor identity by maintaining the proliferation abilities of these cells (Fig. 3A and B) and by inhibiting their differentiation (i.e. progenitor cells express *CDX2* and *HNF1α* but not differentiation genes) (Fig. 3D).

PRC2’s essential role in progenitor cells proliferation was confirmed *in vivo* by monitoring loss of KI67 staining in the intestine of mouse KO for *Eed* (another subunit of this complex) [65, 66]. These studies reveal that progenitor maintenance is mediated by PRC2-dependent inhibition of the *Cdkn2a* cell cycle inhibitor locus (Ink4). This result probably explains the loss of H3K27me3, observed at this locus during enterocyte differentiation in Kazakevych et al.’s study [5] (see Fig. 1B). The KO of *Eed* also induces a loss of ISCs, revealed by diminishing *Lgr5* staining, observed by RNA *in situ* hybridization about 6 weeks after induction of the *Eed* KO, and by loss of the ability to form organoids [66]. The other *in vivo* study [65] does not observe any ISC loss, perhaps due to differences in KO induction. Indeed, in this model (*AhCre;Eed*^*loxp/loxp*^), full recombination requires many successive induction events, and recombination efficiency is lower compared to that observed in Koppens’s work. *Lgr5* expression may thus be compensated by wild-type crypts that are probably overactivated [65, 66].

These studies also illustrate the fundamental role of PRC2 in controlling secretory lineage differentiation. Indeed, the *Eed* KO leads to a huge increase in goblet and enteroendocrine cell numbers, as well as deregulated expression of Paneth cell markers, aberrantly found in the upper part of crypts and in cells expressing goblet cell markers [65, 66]. This ectopic expression validates the importance of H3K27me3 to repress secretory gene promoters in ISCs and enterocytes. Moreover, PRC2 also inhibits secretory lineage differentiation by repressing genes encoding two transcription factors, MATH1 and GFI1 [66]. These complementary mechanisms ensure the robustness of the contribution of Polycomb complexes in lineage establishment.

*In vivo* studies using *Eed* KO mice do not reveal any positive impact on absorptive lineage [65, 66], as opposed to *in cellulo* observations [64, 67]. This difference may be explained by organism difference (Human versus mouse), or through compensation by other factors *in vivo*. However, an obvious explanation could be the masking of absorptive differentiation by the very huge increase in secretory differentiation, not found *in cellulo* due to use of enterocyte-specific differentiation models. The increase, in *Eed* KO mice, of the expression of *Math1*, which is the transcription factor essential and sufficient for choosing the secretory lineage rather than the absorptive one, is in agreement with this hypothesis.

Altogether, these results show the essential role of PRC2 and H3K27me3 in the maintenance of progenitor identity, not only by facilitating their proliferation (inhibition of *Ink4* expression), but also by triggering their choice towards the absorptive lineage (inhibition of *Math1* expression) (Fig. 3D).

### ATP-dependent Remodeling Complexes

ATP-dependent chromatin remodeling complexes contain at least one ATPase subunit, which catalyzes the remodeling reaction, and other subunits acting as cofactors to regulate targeting on specific chromatin regions and to modulate activity. Among the different families of remodeling complexes, the SWI/SNF (also called BAF complex), INO80/SWR and NURD families have been shown to regulate important processes for intestinal cell fate determination.

The SWI/SNF complexes contain BRG1 or BRM ATPases [68]. This family of remodeling complexes primarily exposes local DNA by nucleosome sliding, eviction or unwrapping, thereby facilitating accessibility for DNA binding proteins [69]. Two studies analyze their role in intestinal epithelial homeostasis. Knock-out in the intestine of *Arid1a*, a subunit involved in DNA interaction, leads to loss of ISCs and thus to decreased tissue renewal. Indeed, ARID1a promotes expression of the gene encoding transcription factor SOX9, essential for ISC maintenance [70]. Deletion of the *Brg1 gene*, encoding the ATPase subunit, also causes a drastic decrease in expression of ISC marker genes (*Lgr5, Olfm4, Ascl2*…) [71]. These results show that SWI/SNF complexes are essential for ISC maintenance. The *Brg1* KO likewise leads to a huge increase in secretory cell number, in a Math1–dependent manner [71]. In contrast, *Arid1a* KO diminishes secretory cell number *via* an uncharacterized mechanism [70]. Interestingly, these results show that Brg1 and Arid1a exert opposite roles on secretory lineage commitment. It is tempting to speculate that this is due to antagonistic effects of different SWI/SNF complexes. It would clearly be of interest to analyze the role of Brm, the second ATPase of the SWI/SNF family, in these processes, as well as to characterize the composition of SWI/SNF complexes in the various intestinal lineages.

BRG1-containing SWI/SNF complex can also act as an effector of transcription factors to regulate gene expression. Indeed, BRG1 interacts with β-Catenin [72] and CDX2 [73]. In both cases, the chromatin remodeling activity of BRG1 is essential, leading to a local increase in DNA accessibility, and to the *in cellulo* expression of β-Catenin- and CDX2-target genes (encoding *c-MYC*, *c-MYB* for β-Catenin; *DLL1*, *AXIN2*, *CYP26A1* for CDX2) [72, 73]. Altogether, these results suggest a dual role for SWI/SNF complexes in controlling intestinal cell fate and in mediating the activity of specific transcription factors.

The Tip60/p400 complex, belonging to the INO80/SWR family is a major regulator of the Wnt pathway and controls proliferation of normal and colorectal cancer cells [59]. Maintenance of the ratio between Tip60 and p400 is essential for correct proliferation control. Indeed, as discussed before, Tip60 loss leads to increased proliferation and colorectal preneoplastic lesions, whereas depletion of p400 reverses this phenotype. The Wnt pathway appears to be a major effector of these two chromatin modifiers, which actually regulate it by two different mechanisms: Tip60 inhibits β-Catenin acetylation, whereas p400 promotes expression of Wnt target genes and also Wnt modulators (PORCN and FZD2) [59]. However, it remains to be seen whether p400 complex is recruited by transcription factors of the Wnt pathway (β-Catenin or TCF4) or not. Moreover, SCRAP complex, another INO80/SWR complex, exerts a major role in intestinal homeostasis through H2A.Z histone variant incorporation [37], itself involved in intestinal cell proliferation [36, 37]. Thus, it would be interesting to study if p400 complex regulates Wnt pathway activity and proliferation in a H2A.Z-dependent or -independent way.

The Mi-2/NuRD (Nucleosome Remodeling and Deacetylase) complex is a chromatin complex associated with transcriptional repression; it contains subunits with ATP-dependent chromatin remodeling activity (CHD3 or CHD4), or HDAC activity (HDAC1 or HDAC2). The MBD2 subunit allows recruitment of the complex to the DNA through binding to methylated DNA [74], whereas the MBD3 subunit, which contains a DNA-binding domain unable to interact with methylated DNA [74], targets the NuRD complex thanks to its protein-protein binding properties [75].

In intestinal cells, MBD2/NuRD binds to the DNA-methylated promoter of the *Lect2* gene and inhibits its expression. Lect2 being an endogenous inhibitor of the Wnt pathway [76], MBD2/NuRD activates Wnt targets (*Axin2*, *CyclinD2*…) in a context of high Wnt activity (*Apc*^−/−^ mice) [76].

In addition, Aguilera and collaborators found that MBD3 allows interaction of NuRD with the transcription factor c-jun in intestinal cells [75]. Through its binding to c-jun, MBD3/NuRD inhibits expression of c-jun-target genes, like *Lgr5*, *CyclinD1* or *c-jun* itself, when the JNK pathway is inactive. Activation of the JNK pathway leads to c-jun phosphorylation, loss of MBD3/NuRD binding and thus expression of c-jun target genes. This mechanism is essential for controlling proliferation in intestinal crypts but has no effect on secretory differentiation [75]. Finally, there are also indications that NuRD can regulate processes related to the Notch pathway (stemness, proliferation, lineage choice): in colorectal cancer stem cells, the NuRD complex is indeed recruited by the transcription factor PROX1 to inhibit *Notch1* expression [77] and to enforce the undifferentiated stem-like phenotype of these cells.

Altogether, these results (summarized in Fig. 3A-E) demonstrate the pleiotropic roles of chromatin remodeling complexes in the regulation of processes important for intestine homeostasis.

### DNA Methylation

Cytosine methylation in CG dinucleotides (CpG) is a DNA mark known to repress transcription when it occurs at promoters enriched in CpG (CpG islands). It is very stable and can be transmitted through somatic cell divisions. However, in specific context, this mark can be dynamic in order to regulate biological processes [78]. For example, huge modifications in DNA methylation patterns are associated with primordial germ cell (PGC) establishment and early embryogenesis [79]. Moreover, even though DNA methylation is not essential for ES maintenance, it is necessary for Embryonic Stem cell (ES) differentiation. DNA methylation is actually essential for repression of pluripotency genes during the establishment of transcriptional programs [80].

DNA methylation is deposited by DNMT (DNA methyltransferase) enzymes, with DNMT1 as the methyltransferase responsible for maintaining DNA methylation during cell division, and DNMT3 proteins mediating *de novo* DNA methylation [81]. Removal of DNA methylation can be passive, with progressive dilution along cell division, but also active thanks to the TET (Ten-eleven translocation) proteins. These proteins allow indirect DNA demethylation by successive methyl group oxidations, resulting in removal of the modified nucleotide by the Base Excision Repair mechanism [82].

Two studies showed that DNA methylation is mostly static in intestinal epithelium (Kaaij et al., 2013; Kazakevych et al., 2017). The only DNA methylation differences observed during ISC differentiation are the loss of this mark at enhancers of a few genes more highly expressed in differentiated cells [5, 83]. Another study reported more differences during ISC differentiation by modifying the detection thresholds [84]. The authors observed a decrease in DNA methylation at enhancers of differentiation genes, like *Lph, Alpi* or *Krt20*, and an increase at enhancers of ISC genes, such as *Olfm4*. The intestine-specific knock-out of *Dnmt1* leads to a slight increase of crypt size and *Olfm4* expression, suggesting that DNA methylation could participate in controlling stemness features. The *Dnmt1* KO also leads to overexpression of *Dnmt3b*, and not *Dnmt3a*, in the mouse crypts [85]. The double KO of *Dnmt1* and *Dnmt3b* leads to a huge decrease in mice survival, which is not observed with both single KOs. This mortality is associated with the loss of proliferative cells (Ki67^+^ cells) and of tissue renewal [85]. These results show that *Dnmt3b* overexpression compensates for the loss of *Dnmt1* to preserve progenitor proliferation and tissue maintenance.

Altogether, these results show that DNMT1 and DNA methylation are essential for restriction of stemness in the lower part of the crypt and suggest that these actors are important for progenitor proliferation (Fig. 3A and B). The analysis of knock-outs of genes encoding for TET proteins would be very interesting to improve the understanding of the role of DNA methylation dynamics during differentiation process.

## Role of Chromatin in Intestinal Cell Plasticity

In addition to its essential role in the setup of intestinal cell identity, as discussed in the previous section, chromatin is also involved in the control of tissue plasticity. The term “plasticity” describes, in this review, all changes in cell identity which are not directly linked to tissue homeostasis, but induced following environmental modifications or stresses that trigger an adaptive response.

### Chromatin and Progenitor Plasticity

The choice between absorptive and secretory lineages takes place in progenitor cells. It is controlled by the Notch pathway thanks to a phenomenon called “lateral inhibition”. Indeed, in the Transient Amplifying (TA) compartment (i.e., the second third of the crypt, surrounding the ISC), a cell with an active Notch pathway, which corresponds to an absorptive progenitor, inhibits the Notch pathway in its neighboring cells. These cells then express the transcription factor MATH1 to acquire features of secretory lineage progenitors. This phenomenon is coupled with proliferation arrest in these secretory progenitors. In contrast, absorptive progenitors continue to divide and give rise to enterocytes [86–89]. This process, in favor to enterocytes which are thus predominant in number, is essential for digestive physiology of the intestine and ensures its absorptive function.

Kim and collaborators collected absorptive progenitors (Ent-pro) from *Math1-*KO mice [90], thus without secretory cells [91]. They compared them with secretory progenitors (Sec-pro) collected from mice KO for RBPJ, the Notch pathway transcription factor, and in which absorptive cells are depleted [88]. The distinct transcriptomes of these two collected cell types confirm that they are transcriptionally distinguishable from, for example, the overexpression of cell cycle genes (*Myc, Mcm2*…) in Ent-pro, and the overexpression of genes essential for the secretory lineage setup in Sec-pro (*Spdef, Gfi1, Neurod1, Neurog3*…) [90].

However, this binary choice between absorptive or secretory fate can be reversed, if needed, in case of alteration in one lineage [90]. Indeed, surprisingly, the authors observe only weak differences between Ent-pro and Sec-pro concerning the presence of enhancer-specific marks, such as H3K4me2, H3K27ac, or DNA accessibility, assayed using the DHS-seq technique [90], indicating that chromatin, in both progenitor types, could be permissive to conversion from one to the other upon MATH1 activity, which allows by itself activation of a specific secretory transcriptional program. The same lab, a few years later, showed by ATAC-seq (another DNA accessibility assay), a DNA accessibility difference in some regions negative for H3K4me2 [92]. Thus, a major part of chromatin in Ent-pro and Sec-pro could be permissive to conversion by the action of MATH1, whereas some discrete regions could require chromatin remodeling to allow conversion. To confirm the role of permissive chromatin in the conversion potential, it would be of interest to test the effect of ectopic *MATH1* expression on cell transcriptome, in cells which do not express *Math1* initially and which have a chromatin landscape different than Ent-pro or Sec-pro cells (for example, cells from another epithelium).

### Chromatin and Dedifferentiation

Another phenomenon of intestinal plasticity is dedifferentiation of intestinal cells to acquire previously lost ISC features. This process is essential for maintaining the epithelium integrity and thus organism survival [93, 94]. The first study showing this phenomenon, demonstrated that *Dll1*^+^ cells (secretory progenitor cells) are able to replace *Lgr5*^+^ ISCs when these cells are lost, and to give rise to all epithelial cell types [95]. Mechanisms responsible for this dedifferentiation are not fully known, but it can be hypothesized that migration of non-stem cells to a zone enriched in ISC-maintaining signals (i.e., the ISC niche) would be sufficient [94]. This phenomenon, crucial for organism survival under stress conditions, could also be deleterious for the organism. Indeed, it shows the potential of intestinal cells, with appropriate signals, to change their identity and restart proliferation, potentially contributing to hyperplasia and tumorigenesis [96].

Jadhav and colleagues [92] analyzed DNA accessibility by ATAC-seq in ISCs, absorptive progenitors and secretory progenitors. They observe high DNA accessibility in ISCs and Ent-pro, whereas some regions are more accessible in Sec-pro compared to the other cell types (in approximately 30% of tested regions). They also show that differences between Sec-pro and ISCs are lost during the dedifferentiation process of Sec-pro [92]. Thus, the authors hypothesize that Ent-pro, due to their chromatin profile, are easily able to dedifferentiate without chromatin remodeling, compared to Sec-pro. Even though it is well established that both progenitor types are able to dedifferentiate [95, 97–99], whether they have different dedifferentiation frequencies or efficiencies are still unknown.

The role of chromatin modifiers during the dedifferentiation process was addressed in only one study. Chiacchiera and colleagues demonstrated that PRC2, essential for stemness and progenitor identity in homeostatic conditions, is essential for the dedifferentiation abilities of progenitors after *Lgr5*^+^ ISC loss [65].

**Fig. 4:**
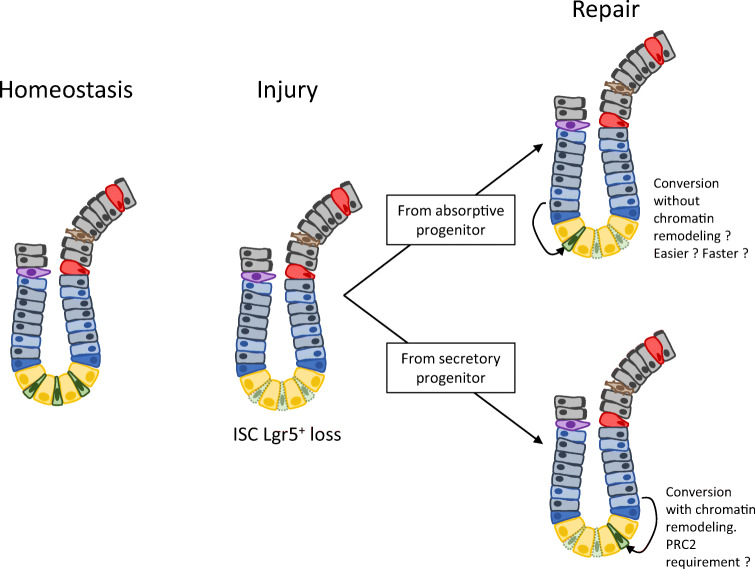
Chromatin remodeling during dedifferentiation. The *Lgr5*^+^ ISC loss is followed by dedifferentiation of progenitor cells to maintain tissue renewal. Dedifferentiation of secretory cells could require global chromatin remodeling, in which PRC2, but also other chromatin modifiers, could be necessary. On the other hand, very similar DNA accessibility profiles between ISC and absorptive cells suggest dedifferentiation from absorptive cells without any global chromatin remodeling. Thus, this way could be easier or faster, increasing the probability of such an event, which could already be favored by the high number of cells engaged in enterocyte lineage

Altogether, these results show that chromatin dynamics are involved in the tissue response to stress (here ISC loss), and that the pre-existing chromatin landscape within each cell type could orientate the choice of the cell type to start dedifferentiation (Fig. 4).

### Chromatin and Adaptation to the Intestine Environment

**Fig. 5:**
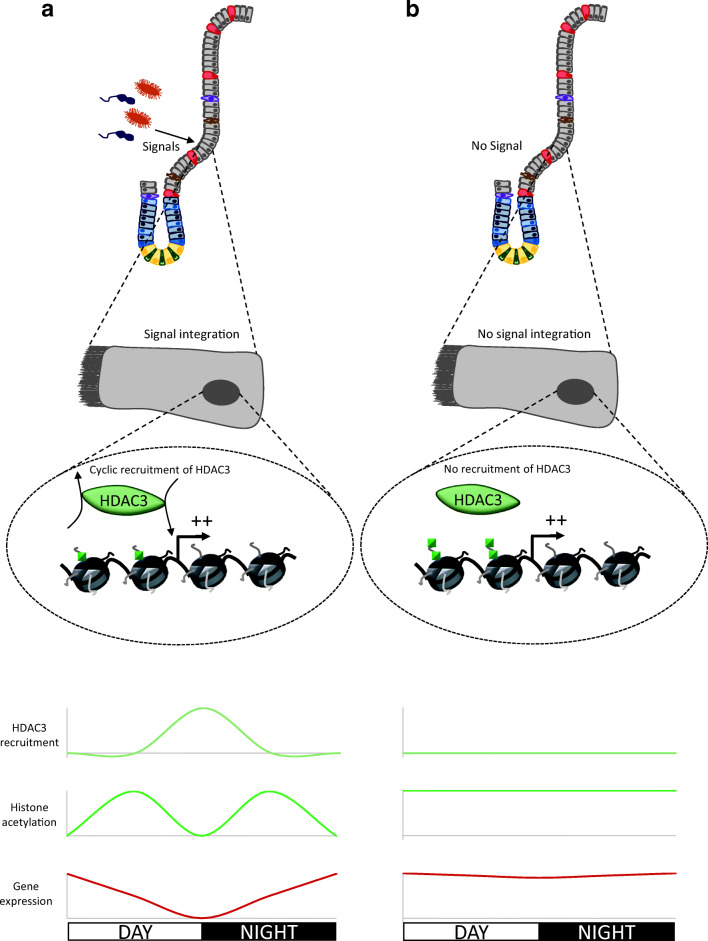
Model of the control of intestinal cell circadian physiology by microbiota ***via*** the chromatin modifications.** A** Intestinal commensal microbiota induce signals promoting intestinal cells to integrate them. At the molecular level, these signals lead to the recruitment of HDAC3 at specific gene promoters, to regulate expression of these genes in a circadian manner, essential for the digestive physiology. HDAC3 is recruited at the end of the day, due to differential microbiota composition and quantity depending of the circadian cycle phase (Thaiss et al., 2016), and then deacetylates histones and thus inhibits gene expression. **B** In germ-free mice, absence of microbiota and their signals lead to loss of HDAC3 recruitment. Histone acetylation and gene expression levels are consistently high during all of the circadian cycle, leading to loss of rhythmicity. Note that graphics schematically summarize HDAC3 recruitment, histone acetylation and gene expression of all genes in this study

Chromatin is undoubtedly essential for the intestine’s adaptation to changes in the outer compartment. Indeed, it was shown that chromatin regulation is required for the epithelium response to commensal microbiota colonization (Fig. 5). Analysis of the chromatin contribution to adaptive responses to other conditions, like pathogenic bacteria infection, or to others environmental factors, like too rich food, pesticide ingestion, drinking water pollution, etc. …, could be of great interest. For example, it was shown that βHB, by inhibiting HDAC, has an impact on intestinal cell physiology [48]. Moreover, βHB is produced by the organism, during long fasting and is found in the circulatory system, thus having an important impact on intestinal chromatin. Moreover, several studies from Goda’s lab have shown that a specific diet (high-fructose or low-fat diet) leads to change in acetylation and methylation on the promoters of genes exhibiting increased of expression with this diet [100–102]. These results suggest that food controls chromatin dynamic to regulate gene expression (encoding here for digestive enzymes) in intestinal epithelial cells, which in turn modify the nutrient intake. This illustrates the feedback loop linking environmental factors to chromatin landscape adaptation to ensure the correct tissue response to such factors.

Intestinal epithelium’s major role is to serve as a front line barrier between the organism and its external environment. This tissue must therefore permanently adapt to environmental cues, such as bacterial colonization. Many studies analyze the host-bacteria relationship, with an emphasis on immune response. However, important studies also show that interaction between commensal microbiota and intestinal epithelium leads to important modifications in the intestinal cell transcriptome (between 2000 and 5000 genes are regulated by microbiota, depending on the intestine segment), including genes other than immunity genes [103, 104]. However, mechanisms involved in this regulation are not fully understood.

Chromatin modifications, thanks to their highly dynamic properties, are obvious candidates for mediating response to bacterial colonization. Despite extensive transcriptome modification, little or no global chromatin changes in the presence or absence of microbiota was observed by analyzing DNA accessibility by DHS-seq [103, 105]. Thus, the authors hypothesize that transcription factors mediate the microbiota impact on host transcriptome by inducing local chromatin modifications. However, later studies, analyzing directly histone post-translational modifications (acetylation, methylation, crotonylation…), revealed major chromatin changes in response to colonization by commensal microbiota [106–109]. All these studies are compiled in a review which discusses microbiota-digestive tract interactions and their consequences on the genome, epigenome and tumorigenesis [110]. These results suggest, by correlating transcriptional and chromatin changes, an essential role for chromatin.

Only one analysis demonstrates the causal role of chromatin in transcriptome regulation in response to microbiota colonization [111]. The authors first observe that the dynamics of H3K9ac and H3K27ac (marks associated with active transcription), during the circadian cycle, are affected by loss of intestinal microbiota. Indeed, the rhythm of acetylation at transcription regulatory regions is lost after microbiota depletion, and the global level of acetylation is increased. This modification of acetylation dynamics correlates with gene expression, which also loses its rhythmicity. Interestingly, KO for the gene encoding HDAC3 leads to the same phenotype with an altered rhythmicity of acetylation and gene expression. Finally, the authors demonstrate that microbiota regulate the dynamics of HDAC3’s presence at the promoters of target genes during the circadian cycle. Altogether, these results show how microbiota regulate gene expression (especially genes involved in nutrient intake) during the circadian cycle by controlling HDAC3 dynamics and consequently histone acetylation [111] (Fig. 5). They show, also for the first time, the causal role of chromatin in modification of the host transcriptome by commensal microbiota.

## Conclusions and Future Directions

### Chromatin Dynamics and Intestine Homeostasis

In this review, we highlight important data uncovering the role of chromatin dynamics in the setup and control of intestinal epithelial homeostasis. Transcriptional programs are central elements in cell identity, and important modifications in gene expression are required for ISC differentiation. By their prominent role in transcription control, chromatin modifications and modifiers are major regulators of these processes, as are transcription factors and signaling pathways (Figs. 2 and 3). Indeed, chromatin dynamics is important at all stages of intestine homeostasis: H2A.Z histone variant (and its regulators), PRC2/H3K27me3 and histone acetylation dynamics are all involved in maintaining undifferentiated cell identity. These redundant functions suggest an important role for chromatin dynamics in the robustness of proliferative cell identity. On the other hand, some histone acetyl transferases are involved in the expression of genes in differentiated enterocytes and in enteroendocrine cells, in the latter in close association with the transcription factor NEUROD1. Interestingly, many studies demonstrate interplay between chromatin marks or modifiers in the regulation of processes like transcription [112–114]. For example, thanks to its ubiquitylation, H2A.Z recruits PRC complexes to inhibit differentiation gene expression in Embryonic Stem Cells [35, 115]. Such cross-talk between chromatin modifications could also operate in intestinal cell fate control.

One of the remaining issues is how the dynamics of these marks and/or modifiers is regulated with respect to signaling pathways. Even though regulation of H2A.Z dynamics is controlled, at least in part, by the Wnt signaling pathway [36] (Fig. 2), data on the regulation of other chromatin factors dynamics are still lacking. Deciphering such links is fundamental for fully understanding how the establishment of chromatin modifications, and thus the genetic program, is linked to intestine physiology. The complex role of SWI/SNF complexes, for example, suggests that their dynamic regulation may have pleiotropic effects.

### Besides Transcriptional Regulation

As described in this review, all data so far point to a function for chromatin modification in intestine homeostasis, via its role in controlling gene regulation. However, chromatin modifications also regulate other processes requiring access to the DNA double helix, such as DNA repair or replication, two processes known to be critical for intestine homeostasis and physiopathology. For example, the control of DNA replication licensing (a step of MCM helicase recruitment at the replication origins, occurring in G1 phase [116]) is essential for intestinal cell fate [117]. Indeed, to inhibit replication, recruitment of helicases is lost during the transition between progenitors and villus cells, suggesting a causal role for this mechanism in the proliferation arrest observed during differentiation [117]. Moreover, the mutational rate in the gut increases with aging, associated to dysfunction of the DNA damage response (DDR) in intestinal stem cells [118]. Finally, knocking out DDR proteins leads to apoptosis loss and mutations accumulation in the intestinal epithelium [119–122]. How chromatin modifications and chromatin modifying enzymes affect these processes in the gut, and whether it is important for intestine development or physiopathology, merits further investigation.

### Chromatin Modification and Epigenetic Mechanisms in the Intestine

Recent data also indicate that chromatin modifications may be the basis of important epigenetic information in the gut. For example, promoters of the 628 genes comprising the adult epithelium signature (i.e., genes expressed at the same level between ISCs and enterocytes, but at a higher level than in embryonic intestinal epithelium) are marked by the active mark H3K4me3 in ISCs as well as enterocytes [5]. This case suggests that H3K4me3 is an epigenetic mark conserved across cell divisions in adult intestinal epithelium. In contrast, several genes, specifically expressed during gut development, are inactive in all cells of adult intestinal epithelium. During intestine development, enhancers of these genes become DNA hypomethylated and marked with H3K4me1 (active enhancers), allowing their expression; in adult epithelium, H3K4me1 is lost but DNA hypomethylation is conserved [123]. Moreover, re-expression of transcription factors specific to developmental stages in mice knocked out for PRC2 is sufficient for reactivation of these enhancers (gain of H3K4me1) [123]. This result suggests that there is an epigenetic memory of DNA hypomethylation at active enhancers, conserved across hundreds of divisions.

Epigenetic mechanisms are also known to be important for the organism’s response to the environment; in the intestinal epithelium, they could play a major role in the physiopathology of the organ, in particular for metabolic diseases, such as obesity. Indeed, more than 80% of obese people regain weight after a restriction diet, in a mechanism known as “Yo-yo dieting” [124]. This adaptation mechanism of the organism, following the loss of weight, is promoted by hormones from several organs [124]. The intestinal epithelium plays an essential role with the production of hormones, such as CCK, PYY or GLP-1 by enteroendocrine cells; interestingly, these hormones remain expressed at a high level more than one year after the restriction diet [125]. These results suggest that this hormone production by the intestinal epithelium, which is maintained over time, is controlled by epigenetic mechanisms, as also suggested for muscles [126].

To what extent such epigenetic mechanisms relying on chromatin modifications are involved in the intestine physiopathology is clearly an open question, whose answer may have important consequences for the therapeutic strategies to fight against metabolic diseases.

### Epigenetic Drugs in Intestine Diseases

Chromatin dynamics is regulated by enzymes which, through their action on chromatin and thus on epigenetic information, may have long term consequences on gene expression. As a result, chromatin modifiers are promising therapeutic targets for many diseases, in particular - but not only - in cancer. Much effort has been made to identify molecules affecting their activities, called “epigenetic drugs”, and many of them are currently used or tested in clinical trials. Given the prominent role of chromatin modifiers in intestine homeostasis, whether such molecules have interesting therapeutic potential in intestine diseases is clearly worth investigating.

For example, understanding how chromatin structure affects cellular plasticity may provide important insights for cancer treatment. Indeed, cancer stem cells (CSCs) are often defined as “fuel” for the tumor, allowing tumor growth and the appearance of differentiated tumor cells [127]. Thus, CSC targeting has been extensively studied as a very interesting therapeutic strategy. Two studies investigated the therapeutic potential of this strategy for colon carcinoma. CSCs were eliminated thanks to the expression of diphtheria toxin receptor [128] or non-dimerized Caspase9 [129] under the control of the promoter for the intestinal stem cell specific gene *Lgr5.* However, tumor growth resumed after the treatment, and the authors hypothesized that this reappearance was due to dedifferentiation of tumor cells, as already observed in healthy intestine, leading to recovery of tumoral growth. An innovative strategy for the fight against gut cancer, but also in others organs, would be to prevent the dedifferentiation process in addition to CSC ablation [94]. Dedifferentiation mechanisms identified in a healthy or tumoral context, could be valuable therapeutic targets in this context. Epigenetic drugs also clearly merit to be tested, given the probable function of chromatin modifiers, as exemplified by the work we described above, showing that PRC2 activity is involved in dedifferentiation after ISC loss [65].

Epigenetic drugs might also be considered for their therapeutic potential against immune-linked disease, such as Intestinal Bowel Disease (IBD), Crohn’s Disease or Ulcerative Colitis. IBD describes idiopathic diseases involving extensive inflammation of intestinal mucosa, due to a strong immune response, which leads to destruction of intestinal structure and function. In addition, to its direct role in controlling immune response (reviewed in [130]), chromatin dynamics could play a role in IBD, thanks to its pivotal role in the control of intestinal cell identity. Indeed, an increasing number of studies shows the importance of epithelial cell dysfunction in the occurrence of IBD, reviewed in [131]. For example, loss of barrier function [132], through dysfunction of tight and adherent junctions, as well as loss of Goblet cells [133], are associated with IBD. Moreover, in mice, *Muc2* Knock-Out leads to spontaneous appearance of chronic colitis [133]. How chromatin deregulations functions in IBD emergence, via altering cell identity, is clearly worth investigating, especially for diseases without a well-known etiology. Furthermore, curing ulcers often associated with IBD requires extensive regeneration of the tissue, in a process called “mucosal healing”. It is clearly a critical step in the treatment of the disease. This healing involves extensive proliferation of the healthy epithelium [131]. Thanks to chromatin dynamics in the proliferative compartments, as we exemplified above, epigenetic drugs could favor a better remission of patients.

Finally, epigenetic drugs could also be valuable in metabolic diseases associated with digestive enzymes misregulation, such as lactose intolerance. Indeed, by targeting chromatin modifiers, epigenetic drugs could restore normal expression of digestive enzymes. They could be particularly valuable for treating Irritable Bowel Syndrome (IBS), which is associated to lactose intolerance and other intestinal dysfunctions (motility, microbiota or immune response disorders) and for which the only available treatment, probiotics, has alone a relatively low efficiency [134].

In this review, we have shown that chromatin dynamics plays an essential role in the homeostasis of the intestinal epithelium, by controlling the establishment and maintenance of cell identities on one hand, but also by playing an essential role in the plasticity of these cells in response to various stresses on the other hand. Finally, these fundamental roles of chromatin modifications could make them major targets in the fight against intestinal diseases such as cancers, IBD and IBS.
